# Surface and intradermal temperature responses of adults to ambient heat stress with and without cooling

**DOI:** 10.14814/phy2.70643

**Published:** 2025-11-04

**Authors:** Zachary J. McKenna, Elizabeth A. Gideon, Erin M. Harper, Taysom E. Wallace, Isa A. Farooqi, Craig G. Crandall

**Affiliations:** ^1^ Institute for Exercise and Environmental Medicine, Texas Health Presbyterian Hospital Dallas Dallas Texas USA; ^2^ Department of Internal Medicine University of Texas Southwestern Medical Center Dallas Texas USA; ^3^ Department of Health, Human Performance and Recreation University of Arkansas Fayetteville Arkansas USA

**Keywords:** measurement, skin, thermal

## Abstract

Accurate measurement of skin temperature is crucial to understanding responses to heat. We tested the hypothesis that intradermal temperature would differ from skin surface temperature during ambient heating with and without fan and water spray (24°C). Nine healthy adults rested in an environmental chamber maintained at 40°C and 15% relative humidity with and without fan, fan + water spray, and water spray. We assessed temperature in the dermal space (intradermal) and on the skin surface (surface) using thermocouple microprobes. In addition, we measured skin temperature using a standard uninsulated thermocouple taped to the skin (standard). During heating intradermal (35.3 ± 0.6°C) was lower than standard (35.9 ± 0.5°C; *p* = 0.005), but not surface (36.0 ± 0.7°C; *p* = 0.056) temperature. With the fan, intradermal (36.9 ± 0.5°C) was lower than both surface (37.8 ± 0.7°C; *p* < 0.001) and standard (37.4 ± 0.5°C; *p* = 0.005) temperature. With fan + water spray, intradermal (32.3 ± 1.5°C) was higher than standard (30.3 ± 2.2°C; *p* = 0.022), but not different from surface (30.8 ± 1.7°C; *p* = 0.125) temperature. With water spray alone, intradermal (31.9 ± 1.4°C) was higher than both surface (30.6 ± 1.7°C; *p* = 0.035) and standard (30.4 ± 1.3°C; *p* = 0.031) temperature. In the heat, skin temperature measured on the surface is higher than intradermal temperature. When cool water was applied to the skin, surface temperature was lower than intradermal temperature.

## 1 INTRODUCTION

Humans have important thermoregulatory mechanisms aimed at maintaining body core temperature within a narrow range. These thermoregulatory responses are driven in part by specialized sensory neurons called thermoreceptors located in the skin, hypothalamus, spinal cord, great veins, and in some abdominal organs (Tansey & Johnson, 2015). These thermoreceptors provide feedback to initiate autonomic mechanisms to initiate either heat conservation (via shivering and cutaneous vasoconstriction) or dissipation (via sweating and cutaneous vasodilation) responses (Cramer et al., 2022). Given that the skin is the largest organ in the human body, and the fact that skin temperature, along with core temperature, directly affects the thermoregulatory and cardiovascular responses to heat stress (Cramer et al., 2022; Shibasaki et al., 2015), it is crucial to understand and accurately measure skin temperature responses during heat stress.

The accurate measurement of skin temperature depends on a variety of factors including measurement site (i.e., location and number of sites measured), ambient conditions (i.e., medium such as air or water and temperature), and metabolic status (i.e., rest vs. exercise) (Taylor et al., 2014). Moreover, there is considerable heterogeneity in regional skin temperatures across the body (Webb, 1992; Zaproudina et al., 2008). Since measuring temperature from the entire skin surface is impractical, mean skin temperature is often estimated using the weighted average from multiple sites (ranging from four to 16 sites). Regarding the measurement methods, it is commonplace to measure skin temperature via thermometry, often with thermocouples or thermistors attached to the skin's surface (MacRae et al., 2018). However, the temperature of such probes affixed to the skin's surface is clearly influenced by other factors such as ambient temperature. That is, while measuring skin surface temperature the sensor system (i.e., the probe) is in contact with the medium of interest (i.e., the skin) and contact with the adjacent environment (e.g., microclimate air, liquids, and clothing).

Since cutaneous thermoreceptors are located beneath the skin's surface, measuring temperature on the surface of the skin may not accurately capture what is being sensed by the cutaneous thermoreceptors thereby driving the thermoregulatory responses to heat stress. Additionally, it is unclear how convection, conduction, and/or evaporation may further modify any potential error when measuring skin temperature. Therefore, we tested the hypothesis that intradermal temperature would differ from skin surface temperature responses during ambient heat stress with and without convection via electric fan use and evaporative/convective cooling via cool water spray.

## 2 METHODS

### 2.1 Participant population

The study protocol and informed consent were approved by the Institutional Review Boards at the University of Texas Southwestern Medical Center and Texas Health Presbyterian Hospital Dallas (STU‐2024‐0636). Participants provided written informed consent and the study was performed in accordance with the principles outlined in the Declaration of Helsinki, including registration in a publicly accessible database (Clinical Trials ID: NCT06593067). Healthy adults were recruited from the greater Dallas‐Fort Worth metropolitan area. Exclusion criteria included: (1) known heart disease or other chronic medical conditions currently requiring regular medical therapy such as cancer, diabetes, uncontrolled hypertension, known hypercholesterolemia, dermatological conditions known to affect thermoregulation, or neurological conditions (e.g., spinal cord injury and peripheral nerve injury); (2) abnormalities detected suggestive of provocable ischemia; (3) undetected cardiac disease or resting left bundle branch block identified on screening electrocardiogram; and (4) current smoker or regularly smoked within the past 3 years.

### 2.2 Experimental protocol

Participants were instrumented in a thermoneutral room before entering a temperature and humidity controlled environmental chamber (CANTROL, Ontario, Canada). The conditions of the chamber were set and maintained at 40°C and 15% relative humidity. While in the chamber, participants were seated upright in a chair. During the first 15‐min control data were collected to determine the skin temperature responses to ambient heat stress (Control: minute 0–15). After 15 min, an electric fan (Dayton, Niles, IL) connected to a variable autotransformer was turned on to 50% of the maximum speed (Fan: minute 15–30). The fan was positioned ~1 m away from the participant's right arm to supply an air velocity of ~2.5 m/s. Beginning at 30 min we sprayed 5 g of 24°C water every 5 min on the instrumented arm (Fan + Water Spray: minutes 30–45). Finally, to determine the isolated effects of water spray, the fan was turned off at 45 min and water spray was continually applied at 5‐min increments to the instrumented arm (Water Spray: minutes 45–60). The order of conditions was selected to avoid the potential for a carryover effect where a prior cooling modality may have influenced the responses during a subsequent cooling modality. For example, we wanted to identify the effects of ambient heat in isolation (e.g., prior to the onset of sweating) on surface and intradermal temperature responses. Further, we wanted to apply water spray last to avoid an effect of residual moisture on the skin that could influence the results for a subsequent cooling modality.

### 2.3 Instrumentation

**FIGURE 1:**
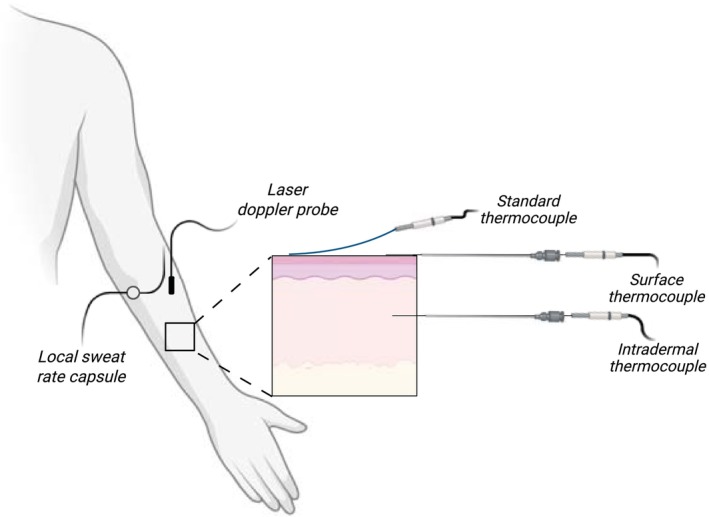
Experimental setup.

Rectal temperature was measured using a general‐purpose temperature probe (Mon‐a‐therm, Mallinckrodt Medical, St. Louis, MO) self‐inserted 10 cm beyond the anal sphincter. We measured skin temperature on the dorsal side of the right forearm using three unique techniques (Figure 1). First, to assess intradermal temperature, a 29‐gauge needle with a thermocouple microprobe (MT‐29/5HT Needle Microprobe) was placed in the dermal space. To facilitate the placing of the thermocouple a 21‐gauge pilot needle was used to penetrate the skin, and the thermocouple was threaded through the lumen of the pilot needle into the intradermal space. The pilot needle was pulled back ~1 cm leaving the temperature sensor component of the 29‐gauge needle in place. Next, an identical 29‐gauge needle with a thermocouple microprobe (MT‐29/5HT Needle Microprobe), threaded through a 21‐gauge pilot needle, was taped on the surface of the skin. The pilot needle was pulled back ~1 cm and care was taken to ensure that the tip of the microprobe, where the temperature sensor is located (i.e., 0.5 mm from the tip), was uncovered and in direct contact with the skin. We chose to use identical methods to assess intradermal and surface temperature so that direct and accurate comparisons could be made between those two probes. Finally, an uninsulated thermocouple was taped to the skin using soft cloth medical tape (3 M™ Medipore™). This approach was used to characterize the standard method used in our laboratory. We chose to use an uninsulated thermocouple to avoid potential alterations in response time. All thermocouples were rated to an accuracy of ±0.1°C per the manufacturer specifications.

Heart rate was obtained from an electrocardiogram (GE Medical Systems, Madison, WI). Skin blood flow was measured using laser Doppler flowmetry (Moor Instruments) on the same forearm as the skin temperature measures. Local sweat rate was measured from the same instrumented forearm using a ventilated capsule technique where anhydrous compressed nitrogen was passed through the capsule at a flow rate of ~300 mL/min. The water content of the nitrogen gas was then measured using capacitance hygrometers (Vaisala, Woburn, WA, USA) and normalized for the area under the capsule.

### 2.4 Validation experiment

To confirm each probe provided accurate measures of temperature, we assessed the temperature responses for each of the employed three techniques by immersing the probes in circulating water maintained at 25, 30, 35, and 40°C (15 min at each temperature).

### 2.5 Data acquisition and statistical analysis

Rectal temperature, intradermal temperature, skin surface temperature, standard skin temperature, laser Doppler flowmetry, and local sweat rate were sampled at 250 Hz (Biopac MP150, Santa Barbara, CA). Continuous data obtained throughout the 60‐min heat exposure are presented for transparency and visualization. To investigate differences between measures of skin temperature, we averaged the temperatures at each site throughout the control (minutes 0–15), fan (minutes 15–30), fan + water spray (minutes 30–45), and water spray (minutes 45–60). Likewise, to interrogate the magnitude of change we calculated the difference from baseline (i.e., time point “0”) and averaged the temperatures at each site throughout the control (minutes 0–15), fan (minutes 15–30), fan + water spray (minutes 30–45), and water spray (minutes 45–60). Averaged data were analyzed using linear mixed effect models with main effects of method (intradermal, surface, and standard), cooling modality (within: control, fan, fan + water spray, and water spray), and the method by cooling interaction. Statistical analyses were performed in GraphPad Prism 10 (GraphPad Software Inc., La Jolla, CA, USA). Significant main effects and interactions were explored using Fisher's LSD test (planned contrasts). Statistical significance was set a priori to *p* < 0.05.

## 3 RESULTS

Results from the validation experiment revealed good agreement (i.e., low variability) between the three thermocouples used to assess skin temperature. The coefficient of variation between the 3 methods was 0.10% at 25°C, 0.11% at 30°C, 0.11% at 35°C, and 0.08% at 40°C. The average temperature recorded throughout the entire 1‐h water bath validation experiment (15 min at 25, 30, 35 and 40°C) was 32.2°C for the intradermal thermocouple, 32.1°C for the surface thermocouple, and 32.1°C for the standard thermocouple.

Nine participants (5 males and 4 females) completed the study. Participant age was 29 ± 4 years (range: 21–33 years); height was 174 ± 8 cm (range: 161–190 cm); weight was 79.9 ± 17.6 kg (61.9–111.8 kg); body surface area was 1.93 ± 0.21 m^2^ (1.65–2.21 m^2^); and body mass index was 27 ± 6 kg/m^2^ (21–39 kg/m^2^). Average conditions within the environmental chamber were 40.6 ± 0.8°C and 12.4 ± 2.9% relative humidity.

**FIGURE 2:**
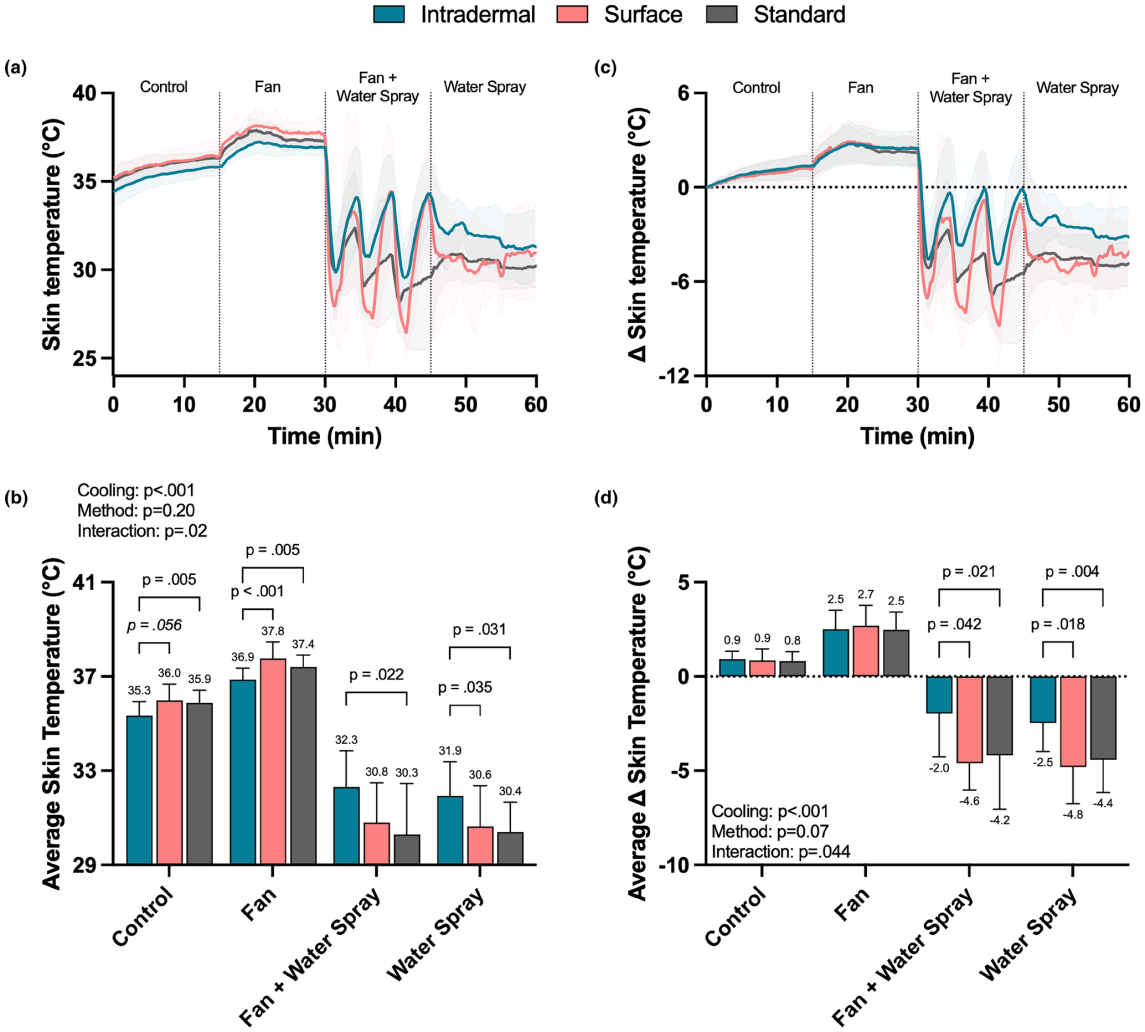
Skin temperature responses to ambient heat stress with and without fan and/or water spray. Fan speed was ~2.5 m/sec, water spray was administered at minutes 30, 35, 40, 45, 50, and 55. Data are shown as mean ± standard deviation. Data in the bottom two panels were analyzed using linear mixed effects models with main effects of cooling, method, and the method by cooling interaction. *N* = 9.

Skin temperature responses to the ambient heat stress with and without the fan and/or water spray are shown in Figure 2. During the first 15 min of heat exposure (i.e., control period) intradermal temperature (35.3 ± 0.6°C) was lower than standard skin temperature (35.9 ± 0.5°C; *p* = 0.005), but not skin surface temperature (36.0 ± 0.7°C; *p* = 0.056). With the fan, intradermal temperature (36.9 ± 0.5°C) was lower than both skin surface (37.8 ± 0.7°C; *p* < 0.001) and standard skin temperature (37.4 ± 0.5°C; *p* = 0.005) measurements. However, there were no differences between the methods for the magnitude of the change from baseline during control and fan exposure (*p* ≥ 0.250; see Figure 2d).

**FIGURE 3:**
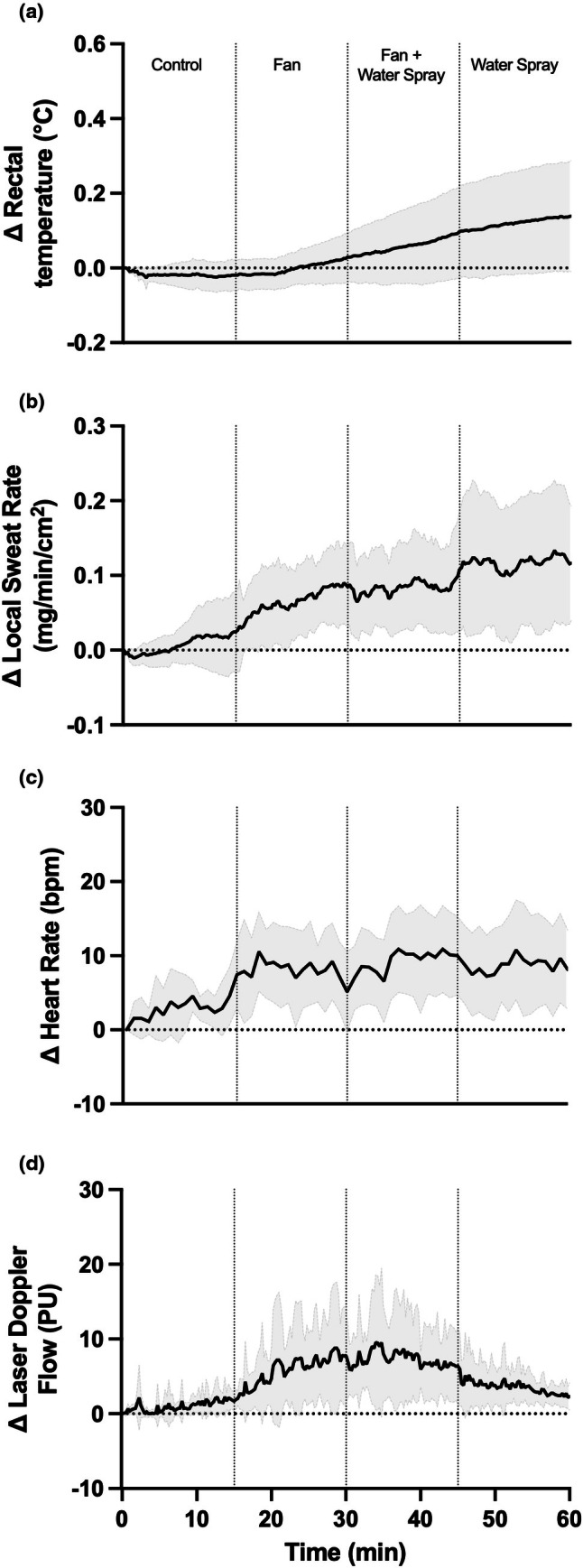
Change in rectal temperature, laser Doppler flowmetry, heart rate, and local sweat rate during heat exposure. Data are shown as mean ± standard deviation. *N* = 9.

With fan + water spray, intradermal temperature (32.3 ± 1.5°C) was higher than standard skin temperature (30.3 ± 2.2°C; *p* = 0.022), but not different from skin surface temperature (30.8 ± 1.7°C; *p* = 0.125). With water spray alone, intradermal temperature (31.9 ± 1.4°C) was higher than both skin surface (30.6 ± 1.7°C; *p* = 0.035) and standard skin temperature (30.4 ± 1.3°C; *p* = 0.031) measures. While less pronounced, these differences persisted when looking at the magnitude of the change from the respective baselines (*p* ≤ 0.042; see Figure 2d). For reference, rectal temperature, laser Doppler flowmetry, and local sweat rate responses throughout the environmental exposure are shown in Figure 3.

## 4 DISCUSSION

The purpose of this study was to compare skin surface and intradermal temperature responses during ambient heat stress with and without electric fan use and/or water spray. Our findings show that in hot ambient conditions (40°C), skin temperature measured on the surface of the skin is higher than intradermal temperature. Further, when cool water spray (24°C) was applied to the skin, surface skin temperature was lower than intradermal temperature. Together, these findings emphasize the influence of environmental conditions on skin temperature measurements and highlight the importance of interpreting surface skin temperature responses to thermal stress with caution.

To evaluate the validity and agreement of the three thermocouples we performed a brief experiment using a circulating water bath set to various temperatures. The results of this experiment showed excellent agreement between the three thermocouples that were used to assess skin temperature, with each thermocouple reading within ±0.1°C and an average coefficient of variation ranging from 0.08% to 0.11% across 25, 30, 35, and 40°C. Thus, the differences in skin temperature reported herein were directly related to the method/site technique (i.e., intradermal or surface) rather than inherent differences in the thermocouples.

We found that surface skin temperatures were slightly higher (~0.6°C) than intradermal temperature during the control and fan periods. This difference was expected given that surface temperatures are influenced by ambient conditions and thus are likely to be higher when measured in the heat. However, we report that the magnitude of the changes from baseline was not different between intradermal and surface temperature measurements during the control and fan periods. This observation was somewhat surprising as we expected surface temperatures to increase to a greater extent than intradermal temperature as the fan delivered warm air onto the skin. The lack of difference in the change values confirms that the main difference observed during the control and fan periods was due strictly to a baseline difference. It is however important to note that the absolute difference between intradermal and surface temperatures was greater with the fan (~0.9°C), suggesting that convective heat gain magnifies the difference between intradermal and skin surface temperatures. The impact of water spray on the measured temperatures was much more pronounced. For example, with water spray, surface temperatures were ~1.0–1.5°C lower than intradermal temperature. These differences persisted when looking at the change from baseline during both the fan + water spray and water spray alone periods. The explanation for these differences is likely due to some combination of convective cooling (via the cool water) and evaporation having a more pronounced effect on surface temperature compared to intradermal temperature. Collectively these findings reemphasize that surface temperature measurements are highly influenced by adjacent environmental conditions.

Practically, these findings may partially explain why previous studies have found a disconnect between the effect of cooling strategies on thermal (i.e., skin and to some extent core temperature) and the cardiovascular response to heat stress. For example, we have previously shown that water spray leads to large and consistent reductions in mean skin temperature (measured from the surface of the skin) in older adults exposed to very hot and dry ambient heat (MacRae et al., 2018). However, these decreases in thermal strain translate into only modest effects on ending heart rate. Since the thermoreceptors are located beneath the skin surface, it is possible that the temperature that is being sensed, and thereby responded to, is not reduced to the same extent relative to what is being measured on the skin surface. Given that the measurement of intradermal temperature is often impractical and slightly more invasive than standard techniques, we suggest that researchers simply consider these implications when measuring and interpreting changes in surface skin temperatures during heat stress. Specifically, when external cooling (e.g., cool water spray) is applied, skin temperature measurements may not entirely reflect what is being sensed by the thermoreceptors.

The conclusions of this study are limited to the employed ambient conditions. Specifically, it is important to consider that the environmental temperature was greater than skin temperatures, and thus it is unclear how these findings might differ if ambient temperature was at or below skin temperatures. Also, we sprayed the forearm with cool (i.e., 24°C) water, which when applied to the entire body has been recently shown to modestly decrease thermal and cardiac strain in older adults exposed to 3 h of ambient heating with and without accompanying activities of daily living (Chaseling et al., 2024; McKenna et al., 2025). Warmer water spray may have a less pronounced effect on skin surface temperatures. We do not know the exact location of the intradermal probe, and while the guide needle was inserted as close to the surface as possible, without measuring the depth we are not entirely certain about the exact probe location in the skin. We did not assess skin temperature from multiple sites. While skin temperature varies across different measurement regions, it is unlikely that these differences would alter the comparison of intradermal and surface temperature responses. The order of conditions was not randomized which may have influenced the results of the study. For example, rectal temperature and local sweat rate appeared to rise throughout the trial, whereas laser Doppler flowmetry fluctuated depending on the cooling condition. Given this observation, we cannot exclude the possibility that any differences in temperature between probe locations for a particular cooling modality may have been influenced by alteration in sweat rate and/or skin blood flow. Finally, we only observed skin temperature responses over the course of 15 min, and we do not know whether the differences in probe temperatures would be altered during longer exposures.

Skin temperature plays an important role in mediating the thermoregulatory and cardiovascular responses to heat in part due to thermoreceptors located beneath the skin's surface. Thus, it is important to obtain accurate measures of skin temperature when considering the thermoregulatory and cardiovascular responses to ambient heat exposures. We show that skin temperature measured on the surface of the skin differs from intradermal temperature due to the influence of adjacent environmental temperature, wind, and skin wetting. Thus, we suggest caution when interpreting changes in skin temperature during heat stress, especially when external cooling (e.g., cool water spray) is applied, as measured skin temperature on the surface may not accurately reflect what is being sensed by the thermoreceptors.

## FUNDING INFORMATION

NIH National Institute of General Medical Sciences (NIGMS): R01GM068865 (CGC); NIH National Heart, Lung, and Blood Institute (NHLBI): F32HL168826 (ZJM).

## CONFLICT OF INTEREST STATEMENT

The authors report no conflict of interest.

## ETHICS STATEMENT

The study protocol and informed consent were approved by the Institutional Review Boards at the University of Texas Southwestern Medical Center and Texas Health Presbyterian Hospital Dallas (STU‐2024‐0636). Written informed consent was provided by all participants prior to their participation in the study.

## Data Availability

Data are available from the corresponding author upon request.

## References

[phy270643-bib-0001] Chaseling, G. K. , Vargas, N. T. , Hospers, L. , Barry, H. , Harwood, A. , Graham, C. , Bartlett, A.‐A. , Debray, A. , Lynch, G. , Capon, A. , Crandall, C. G. , Fiatarone Singh, M. , Mavros, Y. , Bi, P. , Nigam, A. , Chabot‐Blanchet, M. , Gagnon, D. , & Jay, O. (2024). Simple strategies to reduce cardiac strain in older adults in extreme heat. The New England Journal of Medicine, 391, 1754–1756. 10.1056/NEJMc2407812 39504526 PMC12121688

[phy270643-bib-0002] Cramer, M. N. , Gagnon, D. , Laitano, O. , & Crandall, C. G. (2022). Human temperature regulation under heat stress in health, disease, and injury. Physiological Reviews, 102, 1907–1989. 10.1152/physrev.00047.2021 35679471 PMC9394784

[phy270643-bib-0003] MacRae, B. A. , Annaheim, S. , Spengler, C. M. , & Rossi, R. M. (2018). Skin temperature measurement using contact thermometry: A systematic review of setup variables and their effects on measured values. Frontiers in Physiology, 9, 29. 10.3389/fphys.2018.00029 29441024 PMC5797625

[phy270643-bib-0004] McKenna, Z. J. , Atkins, W. C. , Sarma, S. , Gideon, E. A. , Wallace, T. , Farooqi, I. A. , Oldham, Z. R. , & Crandall, C. G. (2025). Evaluating low‐energy cooling strategies on thermal and cardiac strain in older adults exposed to very hot and dry heat with accompanying activities of daily living. Journal of Applied Physiology (1985), 139, 206–218. 10.1152/japplphysiol.00390.2025 PMC12256998 40531347

[phy270643-bib-0005] Shibasaki, M. , Umemoto, Y. , Kinoshita, T. , Kouda, K. , Ito, T. , Nakamura, T. , Crandall, C. G. , & Tajima, F. (2015). The role of cardiac sympathetic innervation and skin thermoreceptors on cardiac responses during heat stress. American Journal of Physiology—Heart and Circulatory Physiology, 308, H1336–H1342. 10.1152/ajpheart.00911.2014 25795714 PMC4451302

[phy270643-bib-0006] Tansey, E. A. , & Johnson, C. D. (2015). Recent advances in thermoregulation. Advances in Physiology Education, 39, 139–148. 10.1152/advan.00126.2014 26330029

[phy270643-bib-0007] Taylor, N. A. S. , Tipton, M. J. , & Kenny, G. P. (2014). Considerations for the measurement of core, skin and mean body temperatures. Journal of Thermal Biology, 46, 72–101. 10.1016/j.jtherbio.2014.10.006 25455943

[phy270643-bib-0008] Webb, P. (1992). Temperatures of skin, subcutaneous tissue, muscle and core in resting men in cold, comfortable and hot conditions. European Journal of Applied Physiology and Occupational Physiology, 64, 471–476. 10.1007/BF00625070 1612090

[phy270643-bib-0009] Zaproudina, N. , Varmavuo, V. , Airaksinen, O. , & Närhi, M. (2008). Reproducibility of infrared thermography measurements in healthy individuals. Physiological Measurement, 29, 515–524. 10.1088/0967-3334/29/4/007 18401069

